# IRSp53 Mediates Podosome Formation via VASP in NIH-Src Cells

**DOI:** 10.1371/journal.pone.0060528

**Published:** 2013-03-26

**Authors:** Tsukasa Oikawa, Hitomi Okamura, Franziska Dietrich, Yosuke Senju, Tadaomi Takenawa, Shiro Suetsugu

**Affiliations:** 1 Laboratory of Cell and Tissue Biology, Keio University School of Medicine, Sinjuku, Tokyo, Japan; 2 Institute of Molecular and Cellular Biosciences, The University of Tokyo, Bunkyo, Tokyo, Japan; 3 University of Duisburg-Essen, Essen, Germany; 4 Kobe University School of Medicine, Kobe, Hyogo, Japan; University of Birmingham, United Kingdom

## Abstract

Podosomes are cellular “feet,” characterized by F-actin-rich membrane protrusions, which drive cell migration and invasion into the extracellular matrix. Small GTPases that regulate the actin cytoskeleton, such as Cdc42 and Rac are central regulators of podosome formation. The adaptor protein IRSp53 contains an I-BAR domain that deforms membranes into protrusions and binds to Rac, a CRIB motif that interacts with Cdc42, an SH3 domain that binds to many actin cytoskeletal regulators with proline-rich peptides including VASP, and the C-terminal variable region by splicing. However, the role of IRSp53 and VASP in podosome formation had been unclear. Here we found that the knockdown of IRSp53 by RNAi attenuates podosome formation and migration in Src-transformed NIH3T3 (NIH-Src) cells. Importantly, the differences in the IRSp53 C-terminal splicing isoforms did not affect podosome formation. Overexpression of IRSp53 deletion mutants suggested the importance of linking small GTPases to SH3 binding partners. Interestingly, VASP physically interacted with IRSp53 in NIH-Src cells and was essential for podosome formation. These data highlight the role of IRSp53 as a linker of small GTPases to VASP for podosome formation.

## Introduction

Reorganization of actin filaments and membranes accompanies many cellular events, such as cell migration, where the leading edge extension and the rearward contraction coordinately occur on the opposite sides of the cell from each other. The leading edge is characterized by the formation of lamellipodia and filopodia, downstream of the functions of the small GTPases Rac and Cdc42, respectively [1]. Lamellipodia and filopodia are well-studied structures, because they can be detected within the cells on a two-dimensional plane such as a culture dish. Cell migration in the three-dimensional extracellular matrix (ECM) is an essential process for tumor cell invasion. Studies with cultured cells suggested that the podosome is the machinery for cell migration in the ECM. Podosomes contain molecules for actin polymerization as well as focal adhesions, and thus are considered to facilitate migration in the ECM [2]–[4]. The existence of podosomes in tissues has been reported recently [5].

Podosomes were first characterized in cells transformed with the Rous Sarcoma virus [6], [7], and the constitutive activation of the Src tyrosine kinase leads to podosome formation [8]. In addition to Src kinase, members of the Rho family of small GTPases, including Cdc42 and Rac, are reportedly essential for podosome formation [9]–[11]. The podosome is a small cylindrical structure rich in actin filaments, typically with a diameter of ∼1 µm or less, and it develops into larger ring-like rosettes, which are thought to be assemblies of small podosomes. Studies of osteoclasts revealed a bundled actin core, surrounded by a branched actin array composed of the Arp2/3 complex and N-WASP, in each podosome [12]–[14].

IRSp53 consists of the I-BAR (inverse BAR) domain, the CRIB motif, the SH3 domain, and the C-terminal variable region by splicing [15]. The I-BAR domain is one of the subfamily domains in the BAR (Bin-Amphiphysin-Rvs) domain superfamily [16]. The BAR domain superfamily proteins deform and sense the membrane that fits each BAR domain structure, and thus have been hypothesized as “sensors” that assemble many binding partners, depending on the membrane curvature [17]–[20]. The BAR domains, including the I-BAR domain, typically fold into helix bundles and form dimer units for membrane binding. The helix bundle is one of the features of small GTPase binding, and some BAR domains reportedly bind to small GTPases directly. Indeed, the I-BAR domain of IRSp53 was initially named the Rac-binding domain (RCB), because it binds to activated Rac [21]. The CRIB motif also binds to small GTPases, and that in IRSp53 specifically binds to Cdc42 [22], [23]. In addition, the SH3 domain of IRSp53 binds to several actin regulators, including Eps8, N-WASP, WAVE2, MENA and VASP [15], [24], [25]. IRSp53 binding to Eps8 facilitates actin filament bundling [26], [27]. Eps8 is also important for Rac activation, and was suggested to regulate podosome formation [28], [29]. IRSp53 reportedly binds to N-WASP for filopodium formation [25], and the role of N-WASP in podosome formation has been well established [14]. In contrast, the role of another Arp2/3 activator that binds to IRSp53, WAVE2, has been well established in lamellipodium formation, but it only plays a marginal role in podosome formation [8], [30].

MENA and VASP belong to the Ena/VASP family proteins, which promote actin filament elongation [31]. In contrast to N-WASP and WAVE2, the elongation mediated by Ena/VASP is not directly related to the Arp2/3 complex. Ena/VASP enhances the assembly of actin monomers at the filament ends. VASP had been shown to cooperate with IRSp53 in filopodia formation [22], [23], [32]. However, the roles of VASP and other members of the Ena/VASP family in podosome formation have not been clarified.

The C-terminus of IRSp53 is spliced to create several isoforms. The shortest form (S) contains the PDZ-domain binding motif, where PDZ-domain containing proteins, such as Lin-7, interact for filopodium formation [33]. The other splicing isoforms (M, L and T) lack the PDZ binding sequence [15]. The splicing variants of IRSp53, the M and L isoforms, have WH2-like motifs at their C-termini, where actin monomer binding was confirmed [34]. The WH2-like motif is also found in the paralogue of IRSp53, IRTKS [35]. The role of T isoform specific amino-acid residues remains unclear.

In this study, we analyzed the function of IRSp53 in podosome formation, and found that it connects small GTPases to VASP in the podosome formation by NIH-Src cells.

## Results

### Positive roles of IRSp53 in podosome formation and invasiveness in Src-transformed NIH3T3 cells

**Figure 1:**
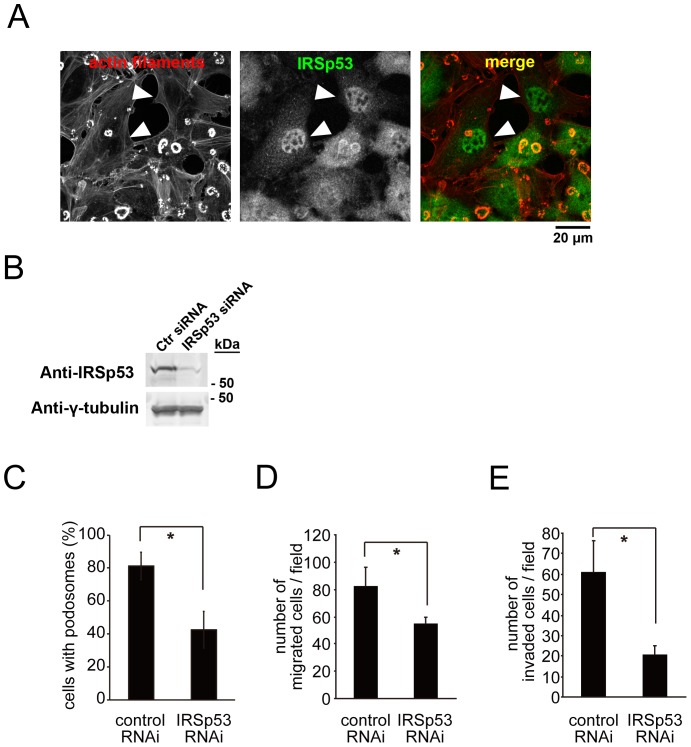
Essential role of IRSp53 in podosome formation, migration and invasion. (A) Localization and involvement of IRSp53 in podosomes. NIH 3T3 cells transformed with constitutively active Src (NIH-Src cells) were transfected with the IRSp53 siRNA vector, and then stained with an anti-IRSp53 antibody (green). Actin filaments were visualized with phalloidin (red). The nuclear staining of IRSp53 represents non-specific signals, because the nuclear localization of GFP-IRSp53 was not obvious (Figure 2). Arrowheads indicate the cells with reduced IRSp53 expression, bearing no or tiny podosomes. Scale bar, 20 µm. (B) NIH-Src cells were transfected with control (Ctr) or IRSp53 siRNA and then cultured for 72 h. The cells were then subjected to immunoblot analyses with the indicated antibodies. (C) NIH-Src cells were transfected with control or IRSp53 siRNA and then cultured for 48 h. The cells were then stained with rhodamine–phalloidin to detect actin filaments, and the percentages of the cells with podosomes were counted. Data are means±SD from three independent experiments. Approximately 100 cells were counted in each determination. *P<0.05 (Student's *t* test). (D and E) The cell migration (D) or invasion (E) assay was performed as described in the materials and methods section, using serum as the chemo-attractant. Data are means±SD from three independent experiments. *P<0.05 (Student's *t* test).

NIH3T3 cells form podosomes when they are transformed with a constitutively active form of Src tyrosine kinase [8]. We used this transformed cell line, NIH-Src cells, as a model for podosome formation. In NIH-Src cells, small dot-like podosomes develop into large “rosette”-like podosomes. To examine the role of IRSp53 in podosome formation, we transfected the cells with the siRNA vector or siRNA oligonucleotides for IRSp53. The amount of IRSp53 was dramatically reduced, as confirmed by cell staining (Fig. 1A, arrowheads) and a western blot analysis (Fig. 1B), and the podosomes disappeared in the cells with reduced IRSp53 expression (Fig. 1A and C). We then examined the migration of the cells using a transwell chamber, with serum as the chemo-attractant. The migration of the cells with reduced IRSp53 expression was significantly impaired (Fig. 1D). Subsequently, because the podosome is a structure that facilitates invasion into the ECM, we examined the invasion activity using a Matrigel invasion chamber. Again, the IRSp53 siRNA treatment dramatically reduced the invasion of the cells through the matrigel (Fig. 1E).

### Redundancy between the IRSp53 splicing variants in podosome formation

**Figure 2:**
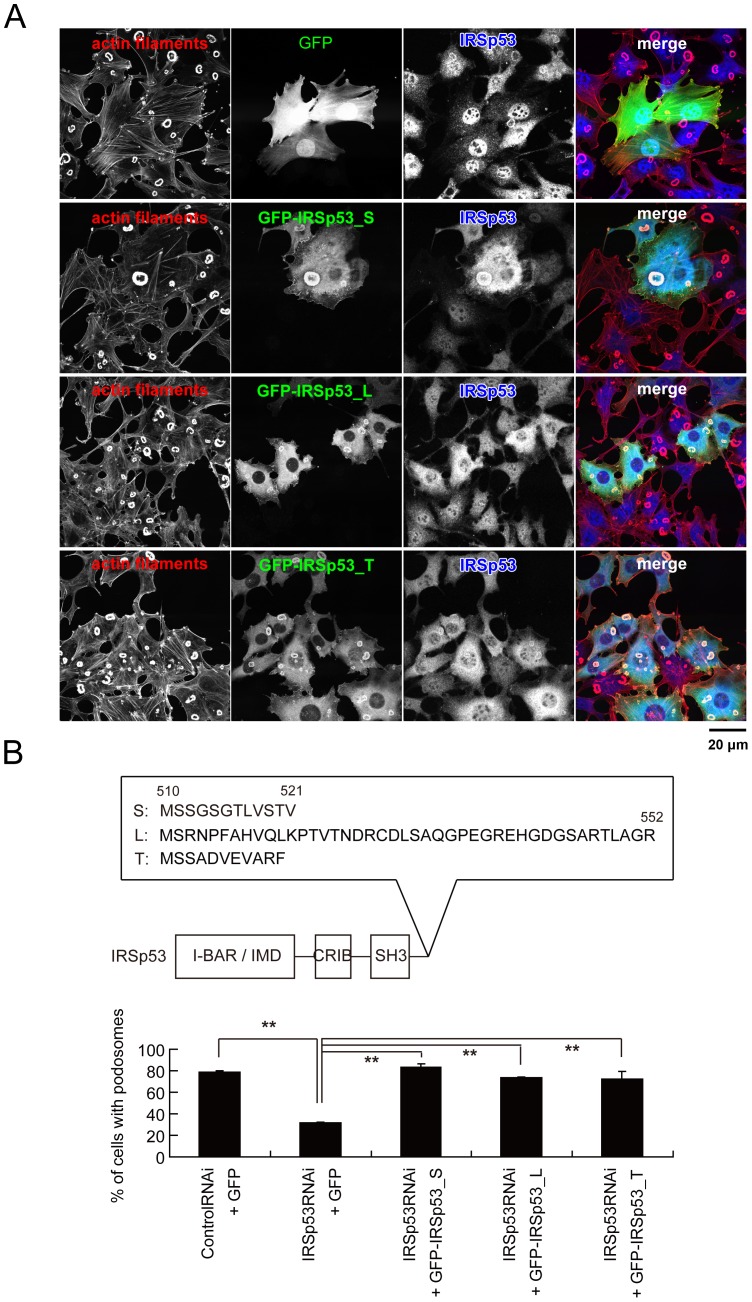
IRSp53 splicing does not affect podosome formation. (A) Restoration of podosome formation by the expression of each IRSp53 splicing isoform. NIH-Src cells were transfected sequentially with the IRSp53 siRNA vector and the expression vectors, and were stained with rhodamine-phalloidin (red) to visualize actin filaments and with anti-IRSp53 (blue). Scale bar, 20 µm. (B) Percentage of cells with podosomes in (A). Data are means±SD from three independent experiments. Approximately 100 cells were counted in each determination. **P<0.01 (Student's *t* test). The C-terminal amino acid residues of the IRSp53 splicing variants are also indicated.

We then examined the specific effect of the IRSp53 siRNA on podosome formation, by restoring IRSp53 expression. The cells were sequentially transfected with the siRNA vector and the expression plasmid for each splicing isoform of human IRSp53 tagged with GFP (Fig. 2). The rescue cDNAs for human IRSp53 were constructed by substituting the nucleotides within the siRNA target, without changing the coding amino acids (see the materials and methods section for details). Each of the splicing isoforms, including (S) with the PDZ binding sequence, the less-characterized isoform (T), and that with the WH2-like motif (L), was able to restore podosome formation, indicating that various C-terminal binding proteins were not involved in podosome formation (Fig. 2).

### Overexpression of the I-BAR domain of IRSp53 abrogates podosome formation

**Figure 3:**
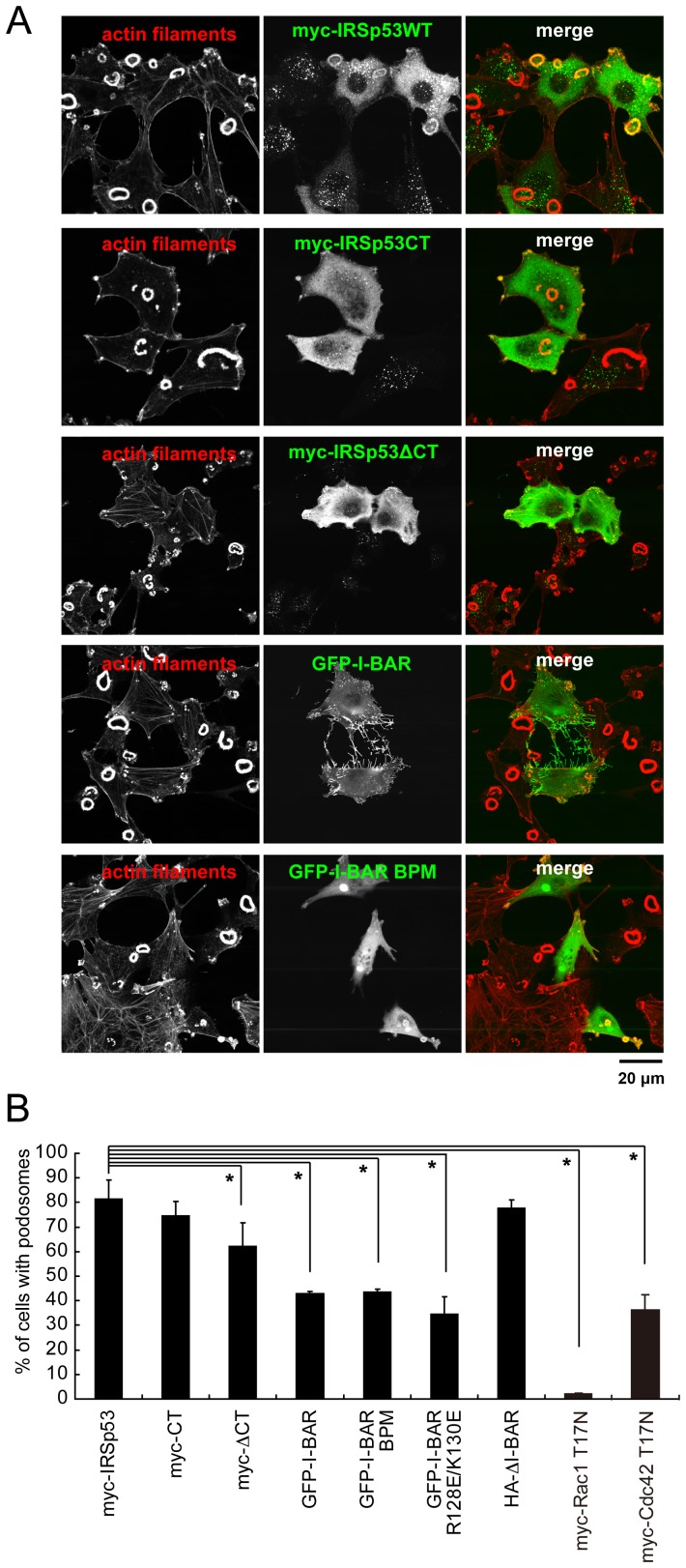
Identification of the domains of IRSp53 required for podosome formation. (A) The podosomes of cells expressing IRSp53 fragments. NIH-Src cells transfected with myc- or GFP-tagged expression vectors were observed by staining with anti-myc (green) or direct fluorescence of GFP, respectively. The cells were also stained with rhodamine-phalloidin (red) to visualize actin filaments. Scale bar, 20 µm. (B) Percentages of cells with podosomes expressing various IRSp53 mutants. The effects of the dominant-negative forms of Rac1 (Rac1 T17N) and Cdc42 (Cdc42 T17N) are also included. Data are means±SD from three independent experiments. Approximately 100 cells were counted in each determination. *P<0.05 versus myc-IRSp53 expressing cells (Student's *t* test). The domain structures of the construct used in this study are indicated in Fig. 5E.

We then used deletion mutants to test which domain of IRSp53 is important for podosome formation (Fig. 3). The overexpression of the wild-type (WT), the C-terminal SH3 domain (CT) of IRSp53, or the fragment without the I-BAR domain had no effects on podosome formation (Fig. 3). On the other hand, the expression of the constructs lacking the C-terminal SH3 domain (ΔCT) or with only the I-BAR domain fragment (I-BAR) suppressed podosome formation (Fig. 3).

**Figure 4:**
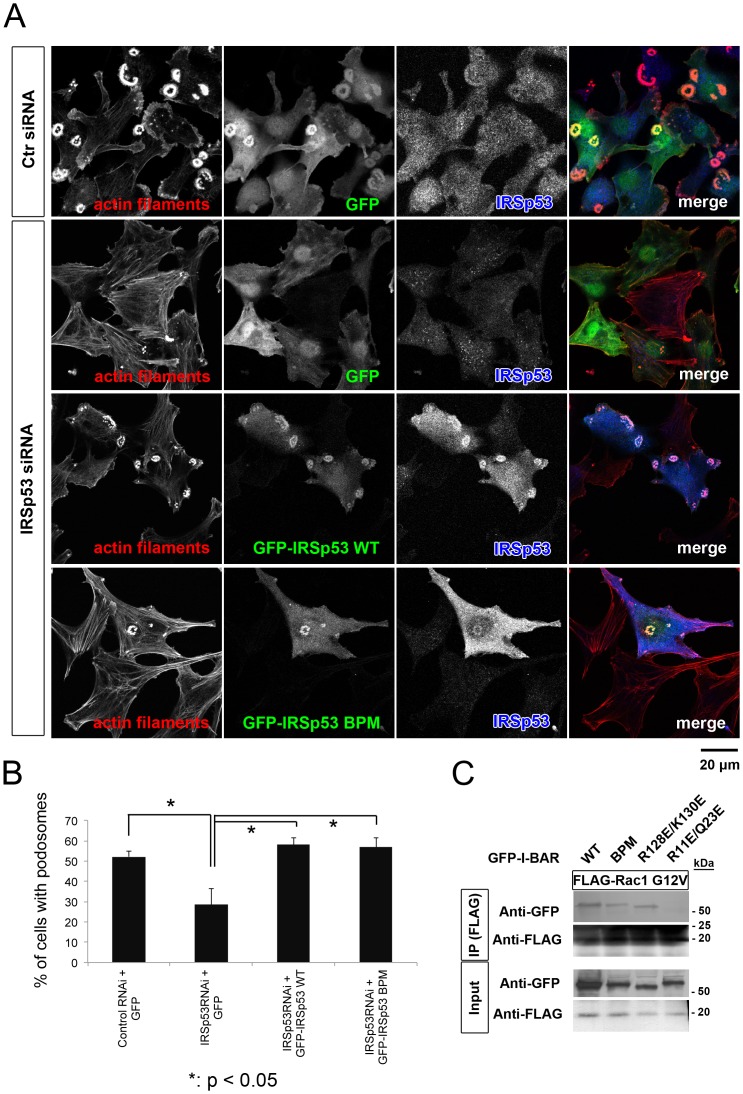
Dispensable role of I-BAR domain for podosome formation. (A) Restoration of podosome formation by the expression of the wild-type (WT) or BPM mutant of IRSp53. NIH-Src cells were transfected sequentially with IRSp53 siRNA and the expression vectors, and were stained with rhodamine-phalloidin (red) to visualize actin filaments and with anti-IRSp53 (blue). Scale bar, 20 µm. (B) Percentage of cells with podosomes in (A). Data are means±SD from three independent experiments. More than 100 cells were counted in each determination. *P<0.05 (Student's *t* test). (C) NIH-Src cells were transfected simultaneously with the FLAG-tagged constitutively active form of Rac (Rac1 G12V) and the GFP-tagged I-BAR domain of IRSp53. The R11E/Q23E mutant of I-BAR, which is defective in Rac binding, was used as a negative control for Rac binding [16]. Anti-FLAG immunoprecipitates were then subjected to immunoblot analyses with the indicated antibodies.

Because the I-BAR domain is known to bind to Rac, actin filaments, and membranes [15], we prepared the mutant I-BAR BPM (basic patch mutant), which is defective in both actin filament and membrane binding, and the I-BAR R128E/K130E mutant, which is selectively deficient in membrane binding [16]. When expressed in NIH-Src cells, the mutants suppressed podosome formation in a similar manner as the wild-type I-BAR (Fig. 3B). Furthermore, IRSp53 with the BPM mutant was able to restore podosome formation in IRSp53 siRNA-transfected cells to the wild-type level (Fig. 4A and B). We then examined the interaction between the BPM and Rac in cells by immunoprecipitation, and found that the I-BAR BPM mutant still interacts with the active form of Rac (Fig. 4C). Therefore, the I-BAR domain was thought to titrate the Rac activity from the podosomes, thus impairing their formation. Actually, the expression of a dominant negative form of Rac1 (Rac N17T) effectively suppressed podosome formation, as reported previously [9]–[11] (Fig. 3B). Taken together, these results suggested that IRSp53 connects Rac signaling to downstream molecules for the actin filament reorganization in podosomes.

### The essential role of VASP in podosome formation

**Figure 5:**
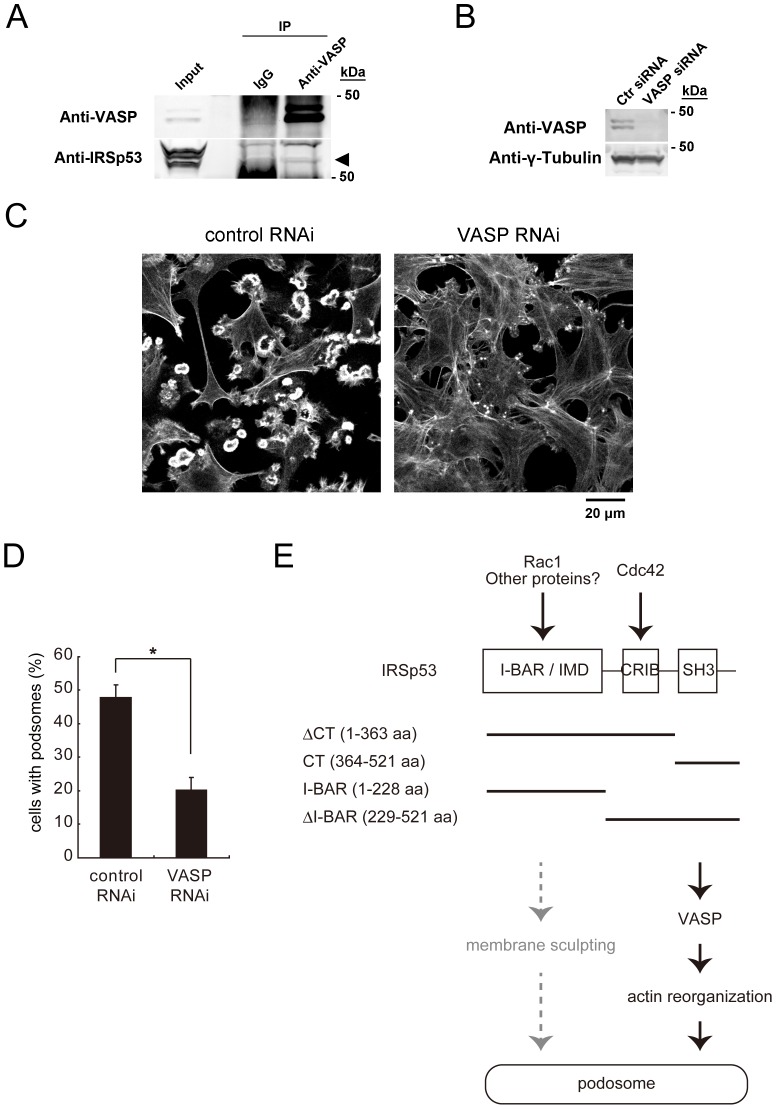
Essential role of VASP in podosome formation. (A) Interaction between IRSp53 and VASP in NIH-Src cells. Lysates of NIH-Src cells were subjected to immunoprecipitation with an antibody to VASP or control immunoglobulin G (IgG). The resulting precipitates, as well as the original cell lysates (Input), were then subjected to immunoblot analyses with the indicated antibodies. The arrowhead shows the band for IRSp53. (B) NIH-Src cells were transfected with control (Ctr) or VASP siRNA and then cultured for 72 h. The cells were then subjected to immunoblot analyses with the indicated antibodies. (C) NIH-Src cells were transfected with control or VASP siRNA, and actin filaments were visualized with phalloidin. Scale bar, 20 µm. (D) Data are means±SD from three independent experiments. More than 100 cells were counted in each determination. *P<0.05 (Student's *t* test). (E) Schematic diagram of the signaling to the actin cytoskeleton through IRSp53 in the podosome. The domain structures of the constructs used in this study are indicated.

We then tried to identify the molecules downstream of IRSp53 that participate in podosome formation. Since the role of VASP in podosome formation has not been characterized so far, we focused on VASP. The immunoprecipitation analysis revealed that VASP interacted with IRSp53 in NIH-Src cells (Fig. 5A). Therefore, we examined the role of VASP in podosome formation. The treatment with VASP siRNA decreased the amount of VASP in NIH-Src cells (Fig. 5B). In these cells, podosome formation was dramatically reduced (Fig. 5C and D). Together, these data suggested that IRSp53 and VASP cooperate in podosome formation (Fig. 5E).

### Discussion

In this study, we established the essential role of IRSp53 in podosome formation in NIH-Src cells. VASP appeared to be the actin regulatory molecule downstream of IRSp53. Interestingly, the membrane deforming ability of IRSp53 was not involved in podosome formation.

Since IRSp53 is a multi-domain protein, the importance of each domain might vary, depending on the cell type. For podosomes in NIH-Src cells, C-terminal splicing of IRSp53 was not essential, suggesting that protein binding at the C-terminal region, which is variable due to splicing, is not crucial. Importantly, the overexpression of the ΔI-BAR mutant did not suppress podosome formation, and thus the role of the I-BAR domain in podosome formation might be marginal. However, it is possible that the ΔI-BAR mutant did not localize properly, and thus could not suppress podosome formation. In contrast, the deletion of the IRSp53 C-terminal region including the SH3 domain suppressed podosome formation, but it was much weaker than the RNAi of IRSp53 (Fig. 3). Therefore, the entire IRSp53 molecule is important for its integrated function in podosome formation, presumably downstream of both Cdc42 and Rac (Fig. 5E).

The strong inhibition of podosome formation by the overexpression of the I-BAR domain highlighted its role in podosome formation. IRSp53 was first proposed to function in actin filament bundling, through its I-BAR domain [36]. However, a recent study demonstrated that IRSp53 I-BAR does not bundle actin filaments under physiological conditions [37]. In this study, the BPM mutant of I-BAR, which is defective in actin filament binding [16], also suppressed podosome formation, suggesting the relatively lower contribution of actin filament binding to the inhibition of podosomes. The I-BAR domain of IRSp53 was subsequently shown to be a subfamily of BAR domain superfamily proteins, in which the I-BAR domain binds to membranes and deforms them into the shape corresponding to the cellular protrusions [16]. In this study, we showed that the overexpression of both the I-BAR domain and its BPM mutant, which is also defective in membrane binding, suppressed podosome formation. In accordance with the overexpression analysis, podosome formation in NIH-Src cells with reduced IRSp53 by siRNA was rescued by the expression of the IRSp53 BPM mutant (Fig. 4). This phenomenon suggested that other binding partners than membranes and actin filaments would be titrated out, resulting in suppressed podosome formation.

The I-BAR domain was first characterized as a Rac-binding domain [21]. Therefore, the titration of Rac could be one of the mechanisms for the suppression of podosome formation by the overexpression of the I-BAR domain, because the BPM retained Rac-binding ability in cells (Fig. 4C). However, the I-BAR domain has several other binding partners with the NPY motif [38]. The NPY motif is present in the Tir protein, which is required for pedestal formation in enterohemorrhagic E. coli (EHEC) infection. Importantly, the NPY sequence is found in a wide range of proteins, such as PI-3 kinase, LIM kinase, Intersectin, and ROCK, suggesting the possibility that the titration of such proteins by I-BAR overexpression can suppress podosome formation [38].

When we consider actin filament organization and podosomes, it would be interesting if IRSp53 functioned with VASP, because podosomes consist of not only branched actin filaments generated by the Arp2/3 complex and N-WASP, but also bundled filaments [13]. VASP does not participate in branched filament formation, but it antagonizes capping protein to promote the straight filaments. Indeed, among the SH3 binding partners of IRSp53, N-WASP directly binds to Cdc42. Therefore, Cdc42 signaling might induce the branched actin filaments through N-WASP and the Arp2/3 complex independently or dependently on IRSp53, and the straight bundled filaments with IRSp53 and VASP dependently on IRSp53.

VASP and MENA play well-established roles in filopodia and lamellipodia formation. However, this is the first study to establish their roles in podosome formation. VASP and MENA are reportedly associated with invasiveness [39]–[43], suggesting that IRSp53 signaling to VASP or possibly to the MENA pathway would be important for cancer cell metastasis *in vivo*.

## Materials and Methods

### Plasmid construction, cell culture and transfection

Mouse NIH cells transformed with Src (NIH-Src cells) were cultured as described previously [8]. The IRSp53 constructs were described previously [16], [21]. For the ΔI-BAR construct, the sequence lacking the N-terminal 250 amino acid residues was cloned with the human IRSp53 cDNA as the template. Stealth siRNA oligonucleotides for mouse IRSp53 were purchased from Invitrogen, and three independent sequences were transfected simultaneously for one gene to reduce the non-specific reduction of mRNA (Figures 1B-E, 4 and 5). For Figures 1A and 2, siRNA was produced from the vector [16], and the rescue cDNAs for human IRSp53 were constructed by substituting the nucleotides (underlined) within the siRNA target to 5′-GGAGCTACAGTACATAGAC-3′, without changing the coding amino acids.

Transfection was performed with the Lipofectamine LTX and PLUS reagents (Invitrogen), according to the manufacturer's protocols. Two days after transfection, the cells were analyzed.

### Antibodies

The anti-IRSp53 antibody was affinity purified from rabbit serum [16]. Anti-γ-Tubulin (SIGMA, mouse monoclonal) and anti-VASP (Cell Signaling, rabbit monoclonal) were purchased.

### Migration/Invasion assay

The NIH-src cells (2.0×10^4^ cells) were plated on a Transwell or BioCoat Matrigel Invasion Chamber (BD Biosciences) for 18 h. siRNA transfection was performed 48 h before plating on the chamber. The chambers were subsequently fixed in 3.7% formaldehyde in PBS for over 30 min. The chambers were then washed in PBS, and the invaded cells were stained with crystal violet. The non-invading cells were manually removed with cotton swabs. After washing the cells with PBS more than three times, the numbers of cells from three different sample points on the lower surface of the chamber were counted.

### Immunofluorescence

For the immunofluorescence analysis, cells cultured on coverslips were fixed with 3.7% formaldehyde or 4% paraformaldehyde in PBS, permeabilized with 0.1% Triton X-100 in PBS for 5 min, and then incubated with primary antibodies for at least 60 min at room temperature. They were then washed with PBS and incubated with Alexa Fluor 488 or 633–conjugated secondary antibodies (Molecular Probes) for 30 min. Cells were also stained with rhodamine–phalloidin (Invitrogen) to detect actin filaments. The cells were finally washed with PBS, mounted on glass slides, and examined with a fluorescence microscope (Olympus FV1000 or Zeiss LSM710).

### Immunoprecipitation

NIH-Src cells were washed with ice-cold PBS and lysed with either radioimmunoprecipitation assay buffer [50 mM Tris-HCl (pH 7.5), 150 mM NaCl, 1 mM EDTA, 1% Triton X-100, 1% sodium deoxycholate, 0.1% SDS] or cell lysis buffer [25 mM Tris-HCl (pH 7.5), 150 mM NaCl, 5 mM EDTA, 2% Triton X-100, 5 mM NaF, 10% glycerol] supplemented with protease inhibitors and phosphatase inhibitors (Sigma). The cell lysates were subjected to immunoprecipitation with agarose bead–conjugated M2 antibodies to FLAG (Sigma) or anti-VASP antibody conjugated to protein A-agarose beads (Pierce), and the bead-bound proteins were analyzed by immunoblotting.

### Statistical analysis

All statistical analyses were performed using Microsoft Excel. Significance was assessed by the Student's *t* test, using data from at least three independent experiments. All images are representative of at least three independent experiments.

## References

[1] Ridley AJ, Schwartz MA, Burridge K, Firtel RA, Ginsberg MH, et al (2003) Cell migration: integrating signals from front to back. Science 302: 1704–1709. 14657486 10.1126/science.1092053

[2] Yamaguchi H, Wyckoff J, Condeelis J (2005) Cell migration in tumors. Curr Opin Cell Biol 17: 559–564. 16098726 10.1016/j.ceb.2005.08.002

[3] Linder S, Aepfelbacher M (2003) Podosomes: adhesion hot-spots of invasive cells. Trends Cell Biol 13: 376–385. 12837608 10.1016/s0962-8924(03)00128-4

[4] Buccione R, Orth JD, McNiven MA (2004) Foot and mouth: podosomes, invadopodia and circular dorsal ruffles. Nat Rev Mol Cell Biol 5: 647–657. 15366708 10.1038/nrm1436

[5] Rottiers P, Saltel F, Daubon T, Chaigne-Delalande B, Tridon V, et al (2009) TGFbeta-induced endothelial podosomes mediate basement membrane collagen degradation in arterial vessels. J Cell Sci 122: 4311–4318. 19887587 10.1242/jcs.057448

[6] David-Pfeuty T, Singer SJ (1980) Altered distributions of the cytoskeletal proteins vinculin and alpha-actinin in cultured fibroblasts transformed by Rous sarcoma virus. Proc Natl Acad Sci U S A 77: 6687–6691. 6256755 10.1073/pnas.77.11.6687 PMC350353

[7] Tarone G, Cirillo D, Giancotti FG, Comoglio PM, Marchisio PC (1985) Rous sarcoma virus-transformed fibroblasts adhere primarily at discrete protrusions of the ventral membrane called podosomes. Exp Cell Res 159: 141–157. 2411576 10.1016/s0014-4827(85)80044-6

[8] Oikawa T, Itoh T, Takenawa T (2008) Sequential signals toward podosome formation in NIH-src cells. J Cell Biol 182: 157–169. 18606851 10.1083/jcb.200801042 PMC2447888

[9] Linder S, Nelson D, Weiss M, Aepfelbacher M (1999) Wiskott-Aldrich syndrome protein regulates podosomes in primary human macrophages. Proc Natl Acad Sci U S A 96: 9648–9653. 10449748 10.1073/pnas.96.17.9648 PMC22264

[10] Morita T, Mayanagi T, Yoshio T, Sobue K (2007) Changes in the balance between caldesmon regulated by p21-activated kinases and the Arp2/3 complex govern podosome formation. J Biol Chem 282: 8454–8463. 17224451 10.1074/jbc.M609983200

[11] Moreau V, Tatin F, Varon C, Genot E (2003) Actin can reorganize into podosomes in aortic endothelial cells, a process controlled by Cdc42 and RhoA. Mol Cell Biol 23: 6809–6822. 12972601 10.1128/MCB.23.19.6809-6822.2003 PMC193918

[12] Luxenburg C, Winograd-Katz S, Addadi L, Geiger B (2012) Involvement of actin polymerization in podosome dynamics. J Cell Sci 125: 1666–1672. 22328507 10.1242/jcs.075903 PMC3346827

[13] Luxenburg C, Geblinger D, Klein E, Anderson K, Hanein D, et al (2007) The architecture of the adhesive apparatus of cultured osteoclasts: from podosome formation to sealing zone assembly. PLoS One 2: e179. 17264882 10.1371/journal.pone.0000179 PMC1779809

[14] Mizutani K, Miki H, He H, Maruta H, Takenawa T (2002) Essential role of neural Wiskott-Aldrich syndrome protein in podosome formation and degradation of extracellular matrix in src-transformed fibroblasts. Cancer Res 62: 669–674. 11830518

[15] Scita G, Confalonieri S, Lappalainen P, Suetsugu S (2008) IRSp53: crossing the road of membrane and actin dynamics in the formation of membrane protrusions. Trends Cell Biol 18: 52–60. 18215522 10.1016/j.tcb.2007.12.002

[16] Suetsugu S, Murayama K, Sakamoto A, Hanawa-Suetsugu K, Seto A, et al (2006) The RAC binding domain/IRSp53-MIM homology domain of IRSp53 induces RAC-dependent membrane deformation. J Biol Chem 281: 35347–35358. 17003044 10.1074/jbc.M606814200

[17] Qualmann B, Koch D, Kessels MM (2011) Let's go bananas: revisiting the endocytic BAR code. EMBO J 30: 3501–3515. 21878992 10.1038/emboj.2011.266 PMC3181480

[18] Suetsugu S, Toyooka K, Senju Y (2010) Subcellular membrane curvature mediated by the BAR domain superfamily proteins. Semin Cell Dev Biol 21: 340–349. 19963073 10.1016/j.semcdb.2009.12.002

[19] Frost A, Unger VM, De Camilli P (2009) The BAR domain superfamily: membrane-molding macromolecules. Cell 137: 191–196. 19379681 10.1016/j.cell.2009.04.010 PMC4832598

[20] Doherty GJ, McMahon HT (2008) Mediation, modulation, and consequences of membrane-cytoskeleton interactions. Annu Rev Biophys 37: 65–95. 18573073 10.1146/annurev.biophys.37.032807.125912

[21] Miki H, Yamaguchi H, Suetsugu S, Takenawa T (2000) IRSp53 is an essential intermediate between Rac and WAVE in the regulation of membrane ruffling. Nature 408: 732–735. 11130076 10.1038/35047107

[22] Govind S, Kozma R, Monfries C, Lim L, Ahmed S (2001) Cdc42Hs facilitates cytoskeletal reorganization and neurite outgrowth by localizing the 58-kD insulin receptor substrate to filamentous actin. J Cell Biol 152: 579–594. 11157984 10.1083/jcb.152.3.579 PMC2195994

[23] Krugmann S, Jordens I, Gevaert K, Driessens M, Vandekerckhove J, et al (2001) Cdc42 induces filopodia by promoting the formation of an IRSp53:Mena complex. Curr Biol 11: 1645–1655. 11696321 10.1016/s0960-9822(01)00506-1

[24] Suetsugu S, Gautreau A (2012) Synergistic BAR-NPF interactions in actin-driven membrane remodeling. Trends Cell Biol 22: 141–150. 22306177 10.1016/j.tcb.2012.01.001

[25] Lim KB, Bu W, Goh WI, Koh E, Ong SH, et al (2008) The Cdc42 effector IRSp53 generates filopodia by coupling membrane protrusion with actin dynamics. J Biol Chem 283: 20454–20472. 18448434 10.1074/jbc.M710185200

[26] Disanza A, Mantoani S, Hertzog M, Gerboth S, Frittoli E, et al (2006) Regulation of cell shape by Cdc42 is mediated by the synergic actin-bundling activity of the Eps8-IRSp53 complex. Nat Cell Biol 8: 1337–1347. 17115031 10.1038/ncb1502

[27] Funato Y, Terabayashi T, Suenaga N, Seiki M, Takenawa T, et al (2004) IRSp53/Eps8 complex is important for positive regulation of Rac and cancer cell motility/invasiveness. Cancer Res 64: 5237–5244. 15289329 10.1158/0008-5472.CAN-04-0327

[28] Goicoechea S, Arneman D, Disanza A, Garcia-Mata R, Scita G, et al (2006) Palladin binds to Eps8 and enhances the formation of dorsal ruffles and podosomes in vascular smooth muscle cells. J Cell Sci 119: 3316–3324. 16868024 10.1242/jcs.03076

[29] Scita G, Nordstrom J, Carbone R, Tenca P, Giardina G, et al (1999) EPS8 and E3B1 transduce signals from Ras to Rac. Nature 401: 290–293. 10499589 10.1038/45822

[30] Suetsugu S, Yamazaki D, Kurisu S, Takenawa T (2003) Differential roles of WAVE1 and WAVE2 in dorsal and peripheral ruffle formation for fibroblast cell migration. Dev Cell 5: 595–609. 14536061 10.1016/s1534-5807(03)00297-1

[31] Bear JE, Svitkina TM, Krause M, Schafer DA, Loureiro JJ, et al (2002) Antagonism between Ena/VASP proteins and actin filament capping regulates fibroblast motility. Cell 109: 509–521. 12086607 10.1016/s0092-8674(02)00731-6

[32] Vaggi F, Disanza A, Milanesi F, Di Fiore PP, Menna E, et al (2011) The Eps8/IRSp53/VASP network differentially controls actin capping and bundling in filopodia formation. PLoS Comput Biol 7: e1002088. 21814501 10.1371/journal.pcbi.1002088 PMC3140970

[33] Crespi A, Ferrari I, Lonati P, Disanza A, Fornasari D, et al (2012) LIN7 regulates the filopodium- and neurite-promoting activity of IRSp53. J Cell Sci 125: 4543–4554. 22767515 10.1242/jcs.106484

[34] Lee SH, Kerff F, Chereau D, Ferron F, Klug A, et al (2007) Structural basis for the actin-binding function of missing-in-metastasis. Structure 15: 145–155. 17292833 10.1016/j.str.2006.12.005 PMC1853380

[35] Millard TH, Dawson J, Machesky LM (2007) Characterisation of IRTKS, a novel IRSp53/MIM family actin regulator with distinct filament bundling properties. J Cell Sci 120: 1663–1672. 17430976 10.1242/jcs.001776

[36] Millard TH, Bompard G, Heung MY, Dafforn TR, Scott DJ, et al (2005) Structural basis of filopodia formation induced by the IRSp53/MIM homology domain of human IRSp53. EMBO J 24: 240–250. 15635447 10.1038/sj.emboj.7600535 PMC545821

[37] Mattila PK, Pykalainen A, Saarikangas J, Paavilainen VO, Vihinen H, et al (2007) Missing-in-metastasis and IRSp53 deform PI(4,5)P2-rich membranes by an inverse BAR domain-like mechanism. J Cell Biol 176: 953–964. 17371834 10.1083/jcb.200609176 PMC2064081

[38] de Groot JC, Schluter K, Carius Y, Quedenau C, Vingadassalom D, et al (2011) Structural basis for complex formation between human IRSp53 and the translocated intimin receptor Tir of enterohemorrhagic E. coli. Structure 19: 1294–1306. 21893288 10.1016/j.str.2011.06.015 PMC4063679

[39] Mouneimne G, Hansen SD, Selfors LM, Petrak L, Hickey MM, et al (2012) Differential remodeling of actin cytoskeleton architecture by profilin isoforms leads to distinct effects on cell migration and invasion. Cancer Cell 22: 615–630. 23153535 10.1016/j.ccr.2012.09.027 PMC3500527

[40] Hu LD, Zou HF, Zhan SX, Cao KM (2008) EVL (Ena/VASP-like) expression is up-regulated in human breast cancer and its relative expression level is correlated with clinical stages. Oncol Rep 19: 1015–1020. 18357390

[41] Han G, Fan B, Zhang Y, Zhou X, Wang Y, et al (2008) Positive regulation of migration and invasion by vasodilator-stimulated phosphoprotein via Rac1 pathway in human breast cancer cells. Oncol Rep 20: 929–939. 18813837

[42] Philippar U, Roussos ET, Oser M, Yamaguchi H, Kim HD, et al (2008) A Mena invasion isoform potentiates EGF-induced carcinoma cell invasion and metastasis. Dev Cell 15: 813–828. 19081071 10.1016/j.devcel.2008.09.003 PMC2637261

[43] Di Modugno F, Iapicca P, Boudreau A, Mottolese M, Terrenato I, et al (2012) Splicing program of human MENA produces a previously undescribed isoform associated with invasive, mesenchymal-like breast tumors. Proc Natl Acad Sci U S A 109: 19280–19285. 23129656 10.1073/pnas.1214394109 PMC3511125

